# Oxytocin does not impact forced-choice recognition memory in an experimental trauma film paradigm with healthy women

**DOI:** 10.3389/fpsyt.2025.1421305

**Published:** 2025-04-03

**Authors:** Tolou Maslahati, Franziska Glogau, Milagros Galve Gómez, Katharina Buchholz, Lisa Dormann, Katja Wingenfeld, Christian Otte, Katharina Schultebraucks, Stefan Roepke

**Affiliations:** ^1^ Department of Psychiatry and Psychotherapy, Campus Benjamin Franklin (CBF), Charité – Universitätsmedizin Berlin, corporate member of Freie Universität Berlin, Humboldt-Universität zu Berlin, and Berlin Institute of Health, Berlin, Germany; ^2^ Department of Psychiatry, New York University (NYU) Grossman School of Medicine, New York, NY, United States; ^3^ Department of Population Health, NYU Grossman School of Medicine, New York, NY, United States

**Keywords:** PTSD, oxytocin, recognition memory, trauma, trauma film paradigm, forced choice memory

## Abstract

**Introduction:**

Traumatic experiences are thought to alter memory acquisition and consolidation. Cognitive models of PTSD suggest that voluntary and involuntary memories after trauma can be independently addressed through interventions. The administration of oxytocin before exposure to a trauma film led to more intrusive (involuntary) memories than placebo. The effect of oxytocin on voluntary memory of the traumatic film, however, remains unclear. The current study aimed to assess whether intranasal oxytocin administration facilitates forced-choice recognition memory after a trauma film paradigm.

**Material & methods:**

We performed a pooled analysis of two randomized, double-blind, placebo-controlled studies (N = 437) to assess the impact of intranasal oxytocin administration on declarative memory. Participants received 24 I.U. of oxytocin, either 40 minutes before a trauma film paradigm or immediately afterward. We applied a forced-choice recognition task seven days after the trauma film paradigm. The task comprised pre-, peri, and post-trauma film scene details.

**Results:**

The administration of oxytocin did not affect recognition performance for any film scene (*F(*2, 401) = .49, *p* = .61). Participants remembered significantly more peri-traumatic film details compared to pre- and post-trauma details (*F(1.72*, 802) = 103.38, *p* <.001).

**Discussion:**

Although the exogenous oxytocin administration before a trauma film has been shown to influence the acquisition of intrusive memories, it does not seem to affect the recognition memory of trauma film details. That aligns with cognitive models of PTSD, suggesting that voluntary and involuntary memory after trauma can be independently addressed through experimental interventions.

## Introduction

During emotionally distressing experiences, hormonal and brain systems influence the acquisition and consolidation of newly acquired memories (1). After traumatic experiences, altered (involuntary and voluntary) memories of the trauma are at the core of Posttraumatic Stress Disorder (PTSD) (2), a mental disorder that has been conceptualized as a disorder of memory (3). Two of the most influential cognitive theories of PTSD, the cognitive model of PTSD (4) and the (revised) dual-representation theory of PTSD (5), suggest that trauma information is processed and stored in two distinct memory systems: involuntary re-experiencing of trauma experiences (intrusive memories) versus voluntary declarative trauma knowledge following elaboration and integration in context and autobiographical memory. Considering the challenges posed by aspects of involuntary memory, it is unsurprising that clinical interventions often focus on mitigating the frequency and intensity of involuntary memory symptoms (6–8). Ideally, interventions target the parts of the memory that trigger emotional and physiological distress but leave the declarative content intact (9). Examining the findings of experimental psychopathology reinforces the notion that interventions may indeed achieve the intended selective impact on both involuntary and voluntary facets of trauma memory. For example, investigations using a visuospatial task in trauma film paradigm studies led to decreased intrusive memories but did not affect recognition memory (10, 11). Protecting voluntary memory of the trauma is crucial for different reasons: voluntary memories are needed to prevent revictimization. Secondly, they are essential for legal documentation and testimony, such as in civil or social claims or criminal proceedings against perpetrators, frequently linked to trauma caused by human actions (10). It remains a crucial challenge to identify vulnerability factors of PTSD to develop preventative treatment for individuals exposed to trauma and further ensure that declarative memory stays unaffected.

Oxytocin, a neuropeptide involved in social cognition, stress regulation, and memory processes, has been discussed as a potential agent for trauma prevention (12). In this context, different mechanisms have been proposed through which oxytocin may influence trauma-related and emotional memory processing.

For one, oxytocin has been shown to interact with the hypothalamic-pituitary-adrenal (HPA) axis (13), which regulates cortisol release, a key stress hormone in memory processes (1). The proposed mechanism underlying this interaction is that oxytocin acts by inhibiting the transcription of the corticotropin releasing hormone and thereby inhibits the HPA axis cascade, leading to a reduced stress response (14). Results regarding the administration of oxytocin in human studies, however, are diverse. While there are confirmatory data showing diminishing effects on the HPA axis [e.g. (15, 16)], a meta-analysis found that oxytocin does not lower HPA axis reactivity per se (17). In their meta-analysis, Cardoso et al. (17) investigated the effect of oxytocin administration on HPA axis activity during laboratory stress tasks. They found that individuals with mental disorders showed a greater diminishing effect than healthy participants. This effect was even greater when the stress task caused a greater cortisol response. Consistent with these findings, we did not observe an effect of nasal oxytocin administration on cortisol release after the trauma, as previously reported (18, 19). Possibly, this absence of an effect could be attributed to the fact that our sample consisted of healthy participants and that, although the trauma film paradigm elicited a significant HPA axis response, it may not have been intense enough to reveal oxytocin-related modulation.

Another proposed mechanism by which oxytocin influences emotional memory processing is its role in neural circuits modulating limbic structures, particularly the amygdala and hippocampus, which are critical for emotional processing and memory consolidation (20). These brain regions, along with the prefrontal cortex, are rich in oxytocin receptors (21), suggesting a direct neuromodulatory role of oxytocin in emotional and memory-related processes. Moreover, oxytocin has been shown to enhance functional connectivity between the amygdala and the prefrontal cortex, a key pathway involved in emotion regulation and top-down control of fear responses (22). However, the effects of oxytocin on memory processing and trauma appear highly context-dependent and influenced by timing and individual differences.

This is reflected in mixed findings regarding its role in trauma-related memory formation: while oxytocin has been shown to prevent PTSD symptoms after traumatic experiences (23), on the one hand, it has also been found to facilitate the acquisition of intrusive memories after a trauma film paradigm (19), on the other. This apparent contradiction is not limited to PTSD-related symptoms but extends to oxytocin’s effects on memory more generally, as research has produced highly inconsistent findings. Specifically, its impact on declarative memory after trauma has yet to be assessed.

Early animal studies led to the assumption that oxytocin generally has amnestic properties on memory processes (24–26). This generalized concept was revised by first acknowledging the influences of dosage and timing on oxytocin effects and then observing memory-enhancing effects of oxytocin [e.g., (27)]. Accordingly, data showed that low doses of oxytocin delivered within one hour post-learning improved consolidation across various memory tasks in animal models, including episodic memory consolidation and retention (28, 29). It was further shown to increase recall of traumatic memory if administered immediately after trauma (foot-shock) (30).

In humans, oxytocin effects on memory processes are also diverse. During acquisition, oxytocin has been found to have memory enhancing as well as memory impairing effects. Accordingly, it improved recall of happy but not of neutral faces (31), it improved recognition of negative social stimuli (32) but also impaired recognition of words in general, irrespective of their valence (33). When administered during consolidation oxytocin improved recognition for negative (34, 35) and neutral but not for happy faces (34). During retrieval oxytocin administration only improved the recognition of social but not of neutral stimuli (36) and also improved the recognition of positive but not negative words (37). Furthermore, oxytocin impaired the ability to identify fearful faces in one study (37) and facilitated it in another (38).

The social salience hypothesis of oxytocin (39) helps to shed light on these contradictory findings. According to this framework, oxytocin does not uniformly enhance or impair memory but rather amplifies the salience of social-emotional stimuli, regardless of their valence (40, 41). As a result, its effects on declarative memory are not straightforward but depend on various factors, including the applied memory task (33, 42), context, and individual differences (43–46). This perspective suggests that oxytocin increases sensitivity to social cues in a way that is shaped by both interindividual factors and situational variables (12). Consequently, generalized statements about the direction of oxytocin’s effects on declarative memory cannot yet be made.

In previous analyses, we have shown that elevated oxytocin levels during the acquisition but not during consolidation increase the number of intrusive memories after exposure to a trauma film paradigm in healthy women (19, 47). To our knowledge, the effect of elevated oxytocin levels during encoding or consolidation of traumatic events on recognition memory has not been investigated yet. Given its role in stress regulation and memory processes, oxytocin’s effects may depend on whether it is administered before or after a traumatic experience. Before an emotional event, oxytocin is thought to enhance salience processing and amygdala reactivity, while post-event administration may facilitate memory consolidation and buffer stress responses. This distinction is particularly relevant, as encoding and consolidation represent two distinct neurocognitive processes that are thought to involve different brain mechanisms and timeframes (48).

The current study investigated the effect of exogenously increased oxytocin levels at the time of encoding (before the trauma film) or consolidation (after the trauma film) of an adverse event on recognition memory after one week in healthy women. To do so, we analyzed data of two experimental, randomized, double-blind, placebo-controlled studies (19, 47), each with a single dose of 24 I.U. nasal synthetic oxytocin (Syntocinon ^®^), using an established trauma film paradigm (49). Following the social salience hypothesis, we expected oxytocin administration before the trauma film to enhance the salience of trauma-related cues and, therefore, enhance memory recognition for peri-traumatic film details compared to placebo. As oxytocin has been shown to act anxiolytically when administered after traumatic events (23) and facilitate memory consolidation when administered after memory acquisition (29) or trauma (30), we also expected oxytocin administration after the trauma film to enhance memory recognition for peri-traumatic film-details.

## Materials and methods

### Participants

The sample consisted of a pooled dataset of two separate studies with a similar design. The only difference between the two studies was the timing of oxytocin administration. Participants (n = 220) of the first study (19) received either 24 I.U. of nasal oxytocin or a placebo before the trauma film. Meanwhile, participants (n = 217) of the second study (35) received 24 I.U. or a placebo immediately after the trauma film. Both studies were completed at the Department of Psychiatry and Neurosciences, Campus Benjamin Franklin, Charité – Universitätsmedizin Berlin. They took place in the same room with an identical setup. Participants were limited to a single participation opportunity and were explicitly advised not to discuss the study design with other potential participants. Consequently, the probability of cohort effects and order effects occurring is minimal.

Due to the sexually dimorphic effect of oxytocin (50), only healthy women who reported female sex were included. In order to control the impact of the menstrual cycle on the endogenous oxytocin concentrations (51), subjects who did not take hormonal contraceptives were tested in the luteal phase only. The menstrual cycle phase of each participant was determined by utilizing self-reported information regarding the start date of their most recent menstruation and the duration of their cycle.

Participants were recruited via university email lists and public postings and received an expense allowance for their participation. Exclusion criteria have been published previously (19, 47). To ensure complete recovery from the trauma film, participants were contacted by phone four weeks after participation and offered psychological care in case of ongoing distress. In the second study (47),, one participant reported persistent intrusive symptoms and subsequently had six counseling sessions with a licensed psychologist. The participant’s distress and intrusive experiences resolved during the aftercare period.

### Procedure

Two randomized, double-blind, placebo-controlled studies with each a single dose of 24 I.U. nasal synthetic oxytocin (Syntocinon ^®^) were applied. The oxytocin and placebo sprays were administered by the experimenters to ensure standardized application. Syntocinon^®^ is a commercially available and standardized nasal spray. The placebo spray contained sodium chloride (0.9%), aqua conservans, chlorobutanol, anhydrous citric acid, and glycerin, but without the active ingredient. To maintain blinding, both sprays were prepared in identical bottles with no distinguishable differences in appearance, smell, or taste. The total volume administered was 0.6 ml. The local ethics committee of Charité – Universitätsmedizin Berlin approved the study protocols of both studies (EA4/162/18; EA4/144/16). Study information was sent to participants at least 24 hours before participation, and informed consent was given upon arrival in the laboratory. Participants were asked not to smoke, do physical exercise, and not to eat or consume caffeine, alcohol, or any beverage other than water one hour before the assessment. Considering cortisol fluctuations (52), the start of every testing was set for 2 p.m.

### Experimental phase

The participants received either 24 I.U. Oxytocin (nasal spray, Syntocinon ^®^) or a placebo preparation (sodium chloride nasal spray) before or immediately after the trauma film. Group allocation occurred randomly in each study. The oxytocin nasal spray and the placebo preparation looked identical to ensure the double-blind design. Peak levels of oxytocin occur within 39 – 51 minutes (53); therefore, the nasal spray was given 40 minutes before the trauma film in the first study. The psychometric assessment, among other things, comprised the Beck Depression Inventory-Revised (BDI-II) (54) and the Childhood Trauma Questionnaire (CTQ) (55). We utilized the BDI-II to identify possible indicators of depressive symptoms. A BDI-II score > 13 was considered clinically relevant and led to exclusion. The CTQ was used to detect any childhood trauma that can increase the risk of PTSD after a secondary trauma in adulthood (56).

After the session, participants filled in a diary to record intrusive memories using the methodology outlined by Holmes and colleagues (57). Detailed results regarding these intrusive memories have been reported elsewhere (19, 47).

### Trauma film paradigm

The applied trauma film is a well-established paradigm (57–59) and was presented in a dark room on a 2 x 2.5m screen. The 14-minute and 40-second clip was extracted from Gaspar Noë’s commercial film “Irreversible” (45). It portrays a distressing sequence in which a woman is subjected to a violent assault, including a brutal rape and physical beating, perpetrated by an unknown assailant in a pedestrian underpass. A trained female investigator remained in the room to monitor adherence to the instructions to ensure that the participants watched the entire scene without any visual (e.g., closing their eyes) or auditory avoidance (e.g., removing their headphones).

### Forced-choice recognition task

We adopted a forced-choice recognition task tailored to the content of the film to measure declarative memory for the trauma film. The film scenes were categorized into three phases: pre-, peri-, and post-trauma. The pre-trauma scene shows how the woman leaves a party, the peri-trauma scene shows how the woman is raped and beaten by a man, and the post-trauma scene shows how the woman’s friend sees her being transported into the ambulance. The initial test construction process is described by Rombold-Bruehl et al. (60), who at first constructed a 24-item version of the recognition test. Four correct and four incorrect statements were formulated for each of the three film sequences. Due to problems with item severity, 11 items were added to the set, resulting in a total of 35 items that participants rated in a dichotomic way. For each statement, one had to choose if the statement was correct *(“correct” vs. “false”)* and how certain one would be about this rating *(“certain” vs. “uncertain”)*. Corresponding to the three film sequences, the recognition test is divided into three item groups, referring to the pre-, peri-, and post-trauma sequences. Twelve items represented the pre-trauma sequence, 12 items described the peri-trauma sequence, and 11 items the post-trauma sequence. For statistical analyses, only 17 out of the 35 items (seven pre-trauma items, four peri-trauma items, six post-trauma items) were used due to problems regarding item severity and item discrimination in our sample, which will be described in more detail in the results section of this paper.

### Statistical analysis

Statistical analyses were conducted using SPSS Version 23.0. Statistical significance refers to a *p*-value < 0.05. Sample characteristics between intervention groups (oxytocin before the trauma film, oxytocin after the trauma film, pooled placebo group) were conducted using Chi-square tests for categorical data and univariate analysis of variance (ANOVA) for continuous data.

We employed a repeated measure mixed design ANOVA to investigate the impact of the intervention (between-subjects factor = oxytocin before the trauma film; oxytocin after the trauma film; placebo) and the film sequence (within-subjects variable with three levels = pre-, peri-, post-trauma scene) on the sensitivity score d´ (d - prime) for each film scene, which served as the dependent variable. According to the signal detection theory of recognition memory (61), the sensitivity score d’ determines a participant’s recognition memory performance, corrected for guessing. Hence, it distinguishes between sensitivity (the ability to answer an item correctly) and response bias (62). In the current study, the participants rated the confidence in their answers as either “certain” or “uncertain”. These ratings were used to calculate *hits* and *false alarms*. According to Maniscalco and Lau (62), a *hit* describes a correct answer with a confidence rating as “certain”, whereas a *false alarm* is an incorrect answer with “certain” confidence. To calculate *d’*, the *false alarm* rate must be subtracted from the *hit* rate. D’ can range from - 1 to + 1. Higher positive *d’* values imply a better recognition performance.

## Results

### Participants characteristics

We incorporated n = 403 out of the total n = 437 enrolled participants in the final analysis. Factors like prolonged interruptions in the experiment resulting from technical issues led to the exclusion of participants. A comprehensive report detailing the inclusion and exclusion of participants in both studies has been previously published (18, 19).

The three groups (oxytocin before the trauma film, oxytocin after the trauma film, and combined placebo group) did not differ significantly in any demographic variable or possible confounding variable presented in **Table 1**.

**Table 1:** Sample characteristics.

Characteristics	Oxytocin before the trauma film (n=104) M (SD) or n (%)	Oxytocin after the trauma film (n = 101) M (SD) or n (%)	Placebo (n = 198) M (SD) or n (%)	Statistics
Age	23.22 (3.39)	24.85 (5.68)	23.97 (4.96)	*F*(400) = 2.95, *p* = .05
Intake of oral contraceptives	37 (35.58)	29 (28.71)	71 (35.86)	*χ* ^2^(2) = 1.68, *p* = .43
Current smoker	25 (24.4)	23 (22.77)	64 (32.32)	*χ* ^2^(2) = 4.02, *p* = .13
BMI	21.88 (2.33)	22.11 (2.95)	21.87 (2.55)	*F*(400) = .32, *p* = .73
CTQ	30.72 (6.33)	31.48 (7.93)	31.94 (8.11)	*F*(400) = .87, *p* = .42
BDI-II	3.53 (4.01)	4.35 (4.21)	4.10 (3.69)	*F*(400) = 1.22, *p* = .29
Participants who had seen the film before	7 (7.29)	9 (9.09)	14 (7.07)	*χ* ^2^(2) = .42, *p* = .81

M, mean; SD, standard deviation; BMI, body mass index; CTQ, childhood trauma questionnaire; BDI-II, Beck depression inventory-revised.

### Mean group differences in trauma film-specific memory

An analysis of the memory measure revealed that some items were insensitive, meaning they were either too easy (more than 80% correct) or too complex (less than 20% correct) to answer. Following the methods by Holmes et al. (57), we only included items correctly answered by > 20% or < 80% of the participants. **Table 2** overviews the difficulty and discrimination of all 35 items. The analyses reported refer to the measure after the invariant items were removed.

**Table 2:** Items of the forced-choice recognition task.

Number	Item	German Items (original)	Sequence	T/F	Item Difficulty
1	“The woman is wearing a dress.”	“Die Frau trägt ein Kleid.”	Pre	T	95.0
**2**	**“The walls are red.”**	**“Die Wände sind rot.”**	**Peri**	**T**	**69.7**
3	“Nobody witnesses the rape.”	“Niemand sieht die Vergewaltigung.”	Peri	F	98.3
4	“The woman is carrying a basket.”	“Die Frau trägt einen Korb bei sich.”	Pre	F	99.3
**5**	**“The man wears a ring.”**	**“Der Mann trägt einen Ring.”**	**Peri**	**T**	**66.9**
6	“When the men leave the party the sun has already risen.”	“Als die Männer die Party verlassen, ist es draußen schon wieder hell.”	Post	F	86.3
7	“The man has a pistol.”	“Der Mann hat eine Pistole.”	Peri	F	96.0
8	“The woman has a purse.”	“Die Frau trägt eine Handtasche bei sich.”	Pre	T	90.0
**9**	**“The boyfriend leaves the party with a group of men.”**	**“Der Freund verlässt die Party in einer Gruppe von Männern.”**	**Post**	**F**	**39.8**
**10**	**“The woman is flagging a taxi.”**	**“Die Frau winkt ein Taxi.”**	**Pre**	**T**	**84.1**
**11**	**“It is a single track road but there is heavy traffic.”**	**“Die Straße ist einspurig, aber viel befahren.”**	**Pre**	**F**	**76.5**
12	“Prostitutes are standing next to the street.”	“An der Straße stehen Prostituierte.”	Pre	T	91.8
13	“The crime scene is cordoned off.”	“Der Tatort ist abgesperrt.”	Post	T	89.6
**14**	**“Leaving the party, one of the men holds a bottle of beer in his hand.”**	**“Einer der Männer hat beim Verlassen der Party eine Bierflasche in der Hand.”**	**Post**	**F**	**38.7**
15	“The woman has blond hair.”	“Die Frau hat blonde Haare.”	Pre	F	93.8
**16**	**“The woman is in a club.”**	**“Die Frau war in einem Club.”**	**Pre**	**F**	**40.9**
17	“The man is wearing a suit.”	“Der Mann trägt einen Anzug.”	Peri	T	86.2
18	“A second man appears in the background.”	“Im Hintergrund erscheint ein zweiter Mann.”	Peri	T	95.8
19	“The friend who wanted to walk her home is leaving the party as the ambulance arrives.”	“Der Freund, der sie nach Hause begleiten wollte, verlässt die Party, als der Krankenwagen da ist.”	Post	T	94.7
**20**	**“The man is wearing a tie.”**	**“Der Mann trägt eine Krawatte.”**	**Peri**	**F**	**65.4**
21	“The man has a briefcase.”	“Der Mann hat eine Aktentasche dabei.”	Peri	F	92.0
22	“The woman lies on a stretcher.”	“Die Frau liegt auf einer Krankentrage.”	Post	T	99.7
**23**	**“One of the men smokes while leaving the party.”**	**“Einer der Männer raucht beim Verlassen der Party.”**	**Post**	**T**	**61.4**
24	“The friends are stopped by an ambulance man.”	“Die Freunde werden von einem Sanitäter aufgehalten.”	Post	F	13.0
**25**	**“The woman wears a necklace.”**	**“Die Frau trägt eine Halskette.”**	**Pre**	**F**	**65.6**
26	“The woman entering the underpass with the man, sees the rape.”	“Die Frau, die mit dem Mann die Unterführung betritt, sieht die Vergewaltigung.”	Peri	F	93.8
**27**	**“The woman carries a cardigan.”**	**“Die Frau hat einen Cardigan dabei.”**	**Pre**	**T**	**15.0**
28	“The knife has a toothed blade.”	“Das Messer hat eine gezackte Klinge.”	Peri	F	87.2
**29**	**“When the woman is standing on the street, a red truck drives by.”**	**“Als die Frau an der Straße steht, fährt ein roter LKW vorbei.”**	**Pre**	**T**	**32.4**
**30**	**“The friend hugs the woman on the stretcher.”**	**“Der Freund umarmt die Frau auf der Trage.”**	**Post**	**T**	**20.1**
**31**	**“The man is wearing a bracelet.”**	**“Der Mann trägt ein Armband.”**	**Pre**	**T**	**39.5**
32	“A woman in a red dress, appears in the background.”	“Im Hintergrund erscheint die Frau mit dem roten Kleid.”	Peri	F	93.3
**33**	**“The friend, who wanted to accompany her home, wears a suit coat, when leaving the party.”**	**“Der Freund, der sie nach Hause begleiten wollte, trägt beim Verlassen der Party ein Jackett.”**	**Post**	**T**	**66.1**
**34**	**“The man sniffs a white bottle.”**	**“Der Mann schnüffelt an einem weißen Fläschchen.”**	**Peri**	**F**	**30.3**
35	“The woman lies on a scratcher and is covered by a white sheet.”	“Die Frau liegt auf einer Krankentrage und ist bis zur Brust mit einem weißen Tuch zugedeckt.”	Post	F	17.9

T, true; F, false; Bold Items were included in the analysis.

**Figure 1:**
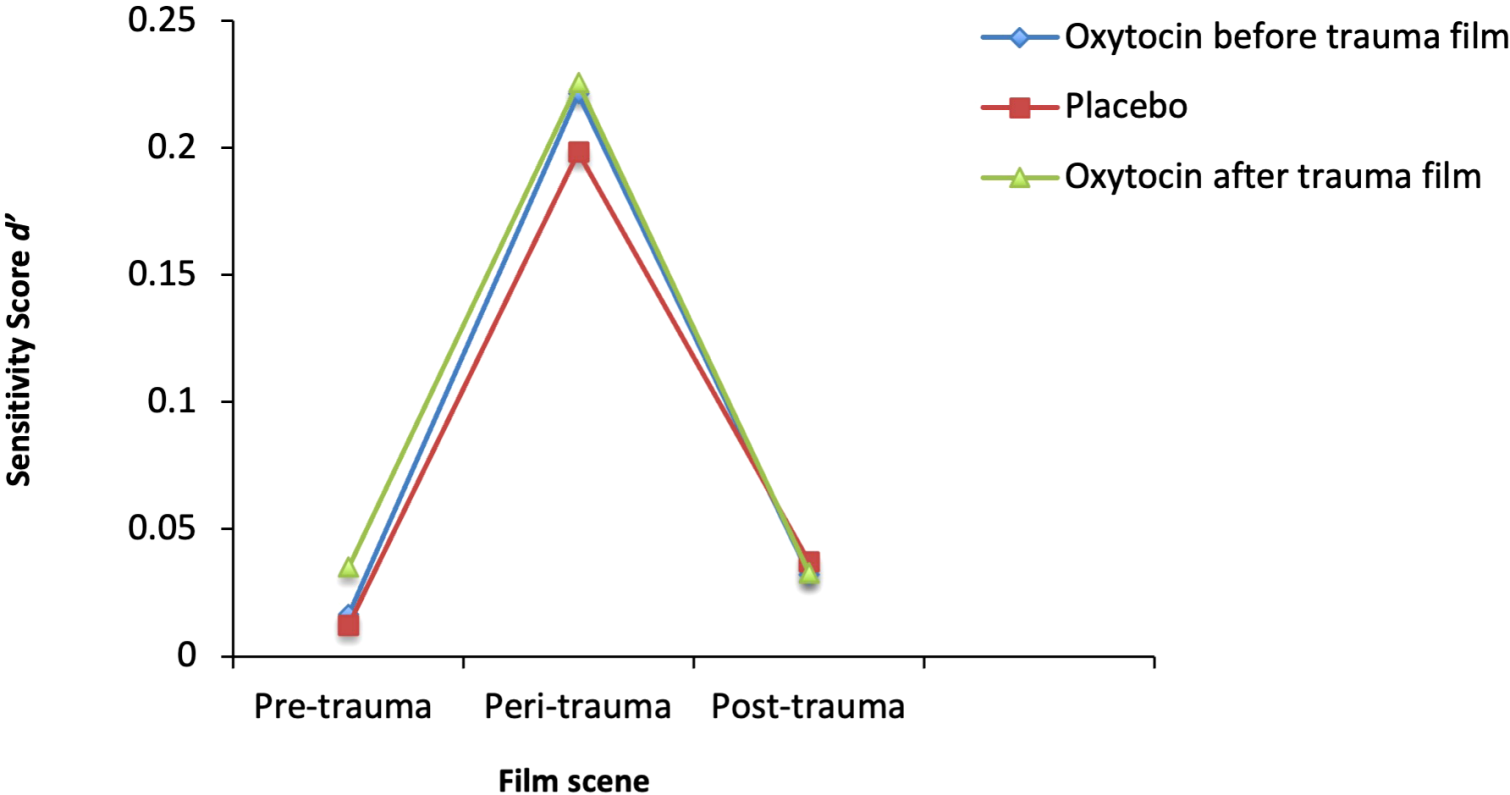
Recognition Performance across the Film Scenes. Mean d´-Scores in the three groups (Oxytocin before the trauma film, Oxytocin after the trauma film, Placebo) for pre-, peri-, and post-trauma film scene memory were assessed after seven days.

The mean hit rates and false alarm rates for the three groups were as follows: For the oxytocin group before the trauma film, the mean hit rate was 2.92 (*SD* = 1.74) and the mean false alarm rate was 2.56 (*SD* = 1.28). For the oxytocin group after the trauma film, the mean hit rate was 3.33 (*SD* = 1.91) and the mean false alarm rate was 2.73 (*SD* = 1.61). For the placebo group, the mean hit rate was 2.89 (*SD* = 1.75) and the mean false alarm rate was 2.67 (*SD* = 1.49). The mean d´scores of the three groups (oxytocin before the trauma film, oxytocin after the trauma film, and combined placebo group) for each film scene (per-, peri-, post-trauma) are shown in **Figure 1**. While there was a significant within-subject factor (film-scene) effect (*F(1.72*, 802) = 103.38, *p* <.001, *ηp2 =* 0.20), there was no significant main effect of oxytocin *F(*2, 401) = .49, *p* = .61, *ηp2 =* 0.01). There was also no significant interaction effect between the treatment and film scene (*F*(3.45,802) = .33, *p* = .83, *ηp2 = 0.00)*. *Post-hoc* tests revealed that peri-traumatic details were remembered significantly more than pre-trauma details (p <.001) and post-trauma details (p <.001).

## Discussion

In this study, we examined the impact of exogenous oxytocin administration before and after a distressing film on forced-choice recognition after one week in healthy women. According to the social salience hypothesis (39), we expected oxytocin administration before the trauma film to enhance trauma-related cues and, therefore, improve memory recognition for peri-traumatic film details. As previous studies about oxytocin have shown anxiolytic effects after traumatic events (23) and enhancing effects on memory consolidation when administered after trauma (30), we also expected oxytocin administration after the trauma film to enhance recognition memory for peri-traumatic film details. Contrary to our hypotheses, we found no effect of either oxytocin before or after the trauma film on recognition memory. We found significant differences in recognition performance regarding trauma film scenes. Participants in all treatment conditions showed a significantly better recognition performance for peri-traumatic film details compared to pre- or post-trauma film details. Meaning, more peri-traumatic details were answered correctly and with more certainty. While pre- and post-trauma film scenes showed mainly neutral content, the peri-trauma film scene consisted of emotional and distressing material showing rape and physical abuse of a woman. That result would align with research on the processing of emotional content and the role of the salience network (63), implying that individuals are more likely to remember information that is emotionally relevant to them (64). Research even suggests that neutral information can be particularly difficult to remember when they appear close to an emotional stimulus (65, 66). However, the result of the current study could also be explained by slightly different difficulty levels of the questions in the applied forced-choice recognition task.

In a previous study, we found that the administration of oxytocin before the trauma film led to more intrusive memories over the following four days (19). Contradictory to our hypothesis, oxytocin administration before the trauma film did not affect recognition memory. An exogenous elevation of oxytocin levels, appears to influence involuntary intrusive memories after a trauma film, while voluntary recognition memory remains unaffected. Correspondingly, a former study with a similar design by our research lab found voluntary and involuntary memory to be independently affected by the manipulation of the noradrenergic system (60, 67). While the activation of the noradrenergic system impacted involuntary memory (intrusions), it did not have an impact on voluntary (recognition) memory. The inhibition of the noradrenergic system on the other hand did only impact voluntary memory and had no effect on involuntary memory (60, 67). These results align with cognitive models of PTSD (4, 68) and research (49, 69), suggesting that voluntary and involuntary memory after trauma can be independently addressed through experimental interventions.

Also, contrary to our hypothesis, oxytocin administration after exposure to the trauma film did not affect recognition memory. That is contradictory to findings showing anxiolytic effects and decreased PTSD symptoms in patients with PTSD (23, 70). Further on, oxytocin has been shown to facilitate memory consolidation when administered after trauma (foot shock) in a rodent study (30). One hypothesis for the missing effect of oxytocin on the consolidation of memories is the administration of only a single dose of nasal oxytocin in our study. A rodent study showed that a single administration of oxytocin directly after a stressor increased contextual fear memory, but a repeated oxytocin administration post-stressor decreased fear generalization (30). Similarly to that study, the aforementioned contradicting seeming study (23) administered oxytocin repeatedly. That indicates that the effects of a single administration may differ from the effects of repeated administration. Administering oxytocin repeatedly might have influenced the outcome of the current study. Another possible hypothesis for the lack of effect in our study may be the degree of distress caused by the trauma film paradigm. The findings from the study conducted by van Zuiden and colleagues (23) suggest that oxytocin does not affect PTSD symptoms per se. Instead, it appears to be only effective in individuals who reported high levels of distress following the trauma. It seems reasonable to consider that the trauma film paradigm administered to healthy individuals may not evoke the same level of distress as actual traumatic events. Consequently, the application of oxytocin in trauma analog studies might not produce a substantial effect on memory consolidation.

### Strength and limitation

The high internal validity of this study due to high experimental control, strict inclusion criteria for the participants, and the inclusion of women only can be highlighted as the study’s main strength. Further strengths of the study are assessing the oxytocin effect before and after the trauma film, examining pre-, peri-, and post-trauma film memory recognition separately, and using a placebo-controlled, double-blind design. As both the menstrual cycle and the intake of hormonal contraceptives have been shown to have an impact on endogenous oxytocin concentrations (51, 71), we considered the intake of hormonal contraceptives and made an effort to control for menstrual cycle by testing naturally cycling women in their luteal phase only. Nevertheless, the luteal phase also shows strong fluctuations in hormonal levels. Future studies may want to choose a narrower subphase of the menstrual cycle. While the homogeneity of our sample is considered a strength in terms of internal validity, it hampers generalization to more vulnerable populations, such as individuals with psychiatric disorders or with previous traumatic experiences. Results can also not be transferred to men, as oxytocin has sexually dimorphic effects potentially due to differences in oxytocin plasma concentrations (40) or different oxytocin expressions in different brain regions of women and men (72). Generalizability may further be restricted as trauma film paradigms are relatively mild stressors compared to actual traumatic events causing PTSD. It remains unclear if conclusions about memory recognition after real trauma can be drawn from the presented data. However, trauma film paradigms offer a valid examination of PTSD risk factors while being ethically justifiable (49).

Next, it is possible that keeping an intrusion diary interfered with memory processing and biased the forced choice recognition task results. Frequent diary entries can enhance memory recognition by reinforcing memory traces through repeated exposure to intrusive memories, which may lead to improved recall over time. One study, for example, showed that individuals who document intrusions experience a heightened sense of immediacy, which can enhance recognition memory performance in tasks requiring quick retrieval (73). At the same time, intrusion diaries may also introduce challenges, such as increased intrusion errors, which may lead to interference, where overlapping memories can confuse recall, potentially complicating the retrieval process and affecting overall memory accuracy (74). Therefore, the potential influence of intrusion diaries on recognition memory should be considered a limitation of the present study, as both memory enhancement and interference effects could have impacted the results. Future research is needed to investigate how intrusion diaries affect recognition memory systematically.

Finally, the applied forced-choice recognition task must be listed as a limitation. We had to exclude 18 out of 35 items to improve item discrimination. While there are valid instruments to examine intrusive memories after trauma film paradigms, there is no standardized and valid measure to examine recognition memory of film details, which has repeatedly been pointed out by different research groups (57, 60). It is, therefore, eminently necessary to construct and validate a standardized memory task for voluntary trauma memory of the applied trauma film paradigm. Different research groups could then use such a task consistently, allowing comparable results across studies. In this regard, it is essential to take various facets of recognition memory into account, given that participants’ accurate responses may be based on recollection, which entails remembering specific details, as well as familiarity, which relies only on the knowledge that an item was present without contextual information (61). It is further essential to pay attention to the type of items included in that kind of questionnaire since the use of suggestive and non-suggestive questions leads to different and sometimes opposing results (75) and to include enough items per category to be able to distinguish between correct recognition and correct rejections (62).

## Conclusion

In summary, exogenous administration of oxytocin either during encoding or during consolidation did not affect the recognition memory after a trauma film paradigm in healthy women. Integrating the findings of previously conducted studies (19, 47), it appears that oxytocin influences the acquisition of intrusive memories after exposure to a trauma film in healthy young women but does not affect the recognition memory of trauma film details. The observation that involuntary and voluntary memory may be influenced separately holds clinical significance. It is important and, at the same time, challenging to find interventions after traumatic experiences that diminish involuntary memories while preserving voluntary memories of the trauma. The latter is crucial, especially in contexts such as legal documentation, testimony, civil or social claims, and criminal proceedings against perpetrators, often associated with trauma resulting from human actions.

## Data Availability

The datasets presented in this article are not readily available because the participants did not agree for the data to be made available publicly. Requests to access the datasets should be directed to TM, tolou.maslahati@charite.de.
